# Cd and Ni transport and accumulation in the halophyte *Sesuvium portulacastrum*: implication of organic acids in these processes

**DOI:** 10.3389/fpls.2015.00156

**Published:** 2015-03-13

**Authors:** Mejda Mnasri, Rim Ghabriche, Emna Fourati, Hanen Zaier, Kebba Sabally, Suzelle Barrington, Stanley Lutts, Chedly Abdelly, Tahar Ghnaya

**Affiliations:** ^1^Laboratoire des Plantes Extrêmophiles, Centre de Biotechnologie de Borj-CédriaHammam-lif, Tunisia; ^2^School of Dietetics and Human Nutrition, McGill University (Macdonald Campus)Sainte Anne de Bellevue, QC, Canada; ^3^Engineering Department of Bioresource, McGill University (Macdonald Campus)Sainte Anne de Bellevue, QC, Canada; ^4^Groupe de Recherche en Physiologie végétale, Earth and Life Institute – Agronomy, Université catholique de LouvainLouvain-la-Neuve,Belgium

**Keywords:** halophytes, phytoremediation, heavy metals, translocation, organic acids

## Abstract

The implication of organic acids in Cd and Ni translocation was studied in the halophyte species *Sesuvium portulacastrum*. Citric, fumaric, malic, and ascorbic acids were separated and quantified by HPLC technique in shoots, roots and xylem saps of plants grown on nutrient solutions added with 50 μM Cd, 100 μM Ni and the combination of 50 μM Cd + 100 μM Ni. Results showed that Cd had no significant impact on biomass production while Ni and the combination of both metals drastically affected plant development. Cadmium and Ni concentrations in tissues and xylem sap were higher in plants subjected to individual metal application than those subjected to the combined effect of Cd and Ni suggesting a possible competition between these metals for absorption. Both metals applied separately or in combination induced an increase in citrate concentration in shoots and xylem sap but a decrease of this concentration in the roots. However, a minor relationship was observed between metal application and fumaric, malic, and ascorbic acids. Both observations suggest the implication of citric acid in Cd, Ni translocation and shoot accumulation in *S. portulacastrum.* The relatively high accumulation of citric acid in xylem sap and shoot of S*. portulacastrum* could be involved in metal chelation and thus contributes to heavy metal tolerance in this species.

## Introduction

As a result of industrial activities, over-fertilization and improper disposal of wastes, pollution of agricultural soils with heavy metals has become increasingly serious throughout the world. These pollutants are characterized by their persistence in the environment and their highly toxic effects to all living organisms (Wong et al., 2002; Rajkumar et al., 2009). Several heavy metals such as Cd, Pb, Hg are non-vital elements and may be toxic even at low concentrations, mainly through their high affinity for S and N atoms in the amino acid side chain (Wei et al., 2003). As a consequence, these elements bind to essential sulfhydryl groups of enzymes or structural proteins, and compete with nutrients such as Ca, Fe, and Mg for transporters in cell membrane (Carrier et al., 2003).

The accumulation of toxic metals in the environment exceeding the threshold level may not only cause visible symptoms of injury in plants, but also imposes serious health hazards to animals and human beings if the contaminated plants are consumed in diet. This type of pollution is more and more frequently generated by power stations, heating systems, metal-working industries, waste incinerators, urban traffic, cement factories or as a by-product of phosphate fertilizers production (Prasad, 1995; Sanità di Toppi and Gabbrielli, 1999). The cleanup of heavy metals contaminated soils is one of the most difficult task for environmental engineering. In most cases, conventional traditional physic–chemical methods are quite expensive and may lead to serious soil alterations (Gardea-Torresdey et al., 2005).

Phytoremediation is based on the use of plants to remove or degrade inorganic and organic pollutants from soils and water. It has been proposed as a promising, environmentally friendly and relatively cheap alternative to classical methods (McGrath et al., 2001). This approach includes distinct strategies such as phytoextraction, phytostabilization, phytovolatilization, phytodegradation, and phytofiltration (Garbisu and Alkorta, 2001). As far as heavy metals are concerned, phytoextraction is especially suitable since those pollutants could not be degraded. The phytoextraction process is based on three essential steps conditioning the final deposition of metals in the shoots. The first one is the pollutants absorption through root system followed by metal transportation from the roots to the shoots and finally the detoxification and sequestration of metals within the shoot tissues.

As far as metal absorption is concerned, several studies demonstrated that plant roots are able to excrete a wide range of organic compounds into their surrounding media to enhance metal availability and facilitate their uptake by roots (Cieslinski et al., 1997; Hall, 2002; Haoliang et al., 2007). These compounds are commonly classified in two categories (i) high molecular weight (HMW) and (ii) low molecular weight (LMW) compounds (Watanabe and Osaki, 2002; Dalla Vecchia et al., 2005; Rascio and Navari-Izzo, 2011). The first one includes mucilage (mainly polysaccharides and polyuronic acid) and ectoenzymes, while the latter mainly consists of organic acids, sugars, phenols and various amino acids, including non-protein amino acids such as phytosiderophores (Marschner et al., 2007).

Organic compounds are also involved in long distance metal transport between roots and shoots (Lasat et al., 2000; Ghnaya et al., 2013). X-ray absorption analysis indeed demonstrated that metal ions are combined with oxygen or nitrogen atoms in the xylem sap which suggests that their translocation might involve organic acids or amino acids (Wei et al., 2007). Cultivation of plants under metal constraints commonly induces important accumulation of low molecular weight organic acids in various plant organs and in the xylem-sap reinforcing the hypothesis that these molecules are involved in root-to-shoot translocation of several metal ions in the form of bound complexes (Tatár et al., 1998; Haydon and Cobbett, 2007; Ghnaya et al., 2013). For example, nickel exists in the form of Ni(II)-citrate complexes in the leaves from Ni-hyperaccumulators species from New Caledonia (Berazaín et al., 2007) while in *Alyssum murale* X-ray experiments also demonstrated that citric acid was the main ligand responsible for long distance transport of nickel (Montargès-Pelletier et al., 2008)

Beside their contribution to heavy metal translocation, organic acids may also be involved in metal detoxification through chelating processes leading to reduction of the free ionic forms of metals which are by far the most toxic forms. Also it was suggested that the build-up in shoot citrate concentrations under HMs exposure could be positively correlated with plant capability to detoxify and accumulate Cd in several plant species (Krämer et al., 2000; Sun et al., 2006; Ghnaya et al., 2013). For example, the Cd-hyperaccumulator *Thlaspi caerulescens* (syn. *Noccaea caerulescens*) synthesizes more organic acids when subjected to Cd^2+^ in order to reduce the reactivity of free Cd^2+^ ions with proteins thus allowing a high accumulation of Cd in the shoots without injury symptoms (Salt et al., 1999; Pence et al., 2000).

*Sesuvium portulacastrum* is a dicotyledonous halophyte belonging to the Aizoaceae family and is commonly known to accumulate large quantities of salts in its above ground tissue. It constitutes a promising plant species for phytoremediation of heavy metal polluted soils (Zaier et al., 2010; Ghnaya et al., 2013) but the precise role of organic acid in the tolerance mechanisms of this species still needs to be confirmed. Moreover, all data available for this species concern plant exposure to one single heavy metal, although polluted sites are frequently contaminated by several contaminants. The aim of this work was therefore to study the relation between the accumulation of cadmium and nickel applied separately or concomitantly and organic acids concentrations in roots, shoots and xylem sap to confirm the possible implication of these compounds in the translocation and sequestration of heavy metals in the halophyte species of *S. portulacastrum*.

## Materials and Methods

### Reagents

All solutions were prepared in MilliQ purified water (Millipore, Molsheim, France). The standard solutions were prepared by appropriate dilution of cadmium and nickel standard solutions (1000 μg L^-1^, Merck, Darmstadt, Germany). All reagents used were of analytical-reagent-grade. Organic acids were obtained from Sigma (St. Louis, MO, USA), and the other reagents were purchased from Merck (Darmstadt, Germany). Stock solutions were prepared by dissolving malic, citric, fumaric, and ascorbic acids in double distilled water and were kept at 4^∘^C. Analytical standard solutions were prepared from these stock solutions by serial dilutions.

### Plant Material and Xylem Sap Collection

*Sesuvium portulacastrum*, was propagated by cuttings from mother plants cultivated in greenhouse. Three cm-long stem segments with one node and two opposite leaves were sampled, sterilized by a 5 min treatment in saturated calcium hypochlorite solution and thoroughly washed with distilled water. They were then placed for 7 d in 1–10th strength aerated Hoagland solution. Rhizogenesis took place after the first week.

The rooted cuttings were transferred for 21 days on an aerated Hoagland’s complete nutrient solution (20 seedlings per treatment) spiked with the appropriate levels of Cd, Ni singly or in combination [control without heavy metals, 50 μM Cd, 100 μM Ni and the combination (50 μM Cd + 100 μM Ni)]. The Hoagland’s solution consisted of 5 mM Ca(NO_3_)_2_, 5 mM KNO_3_, 1 mM KH_2_PO4, 50 μM H_3_BO_3_, 1 mM MgSO_4_, 4.5 μM MnCl_2_, 3.8 μM ZnSO_4_, 0.3 μM CuSO_4_ and 0.2 μM (NH_4_)_6_Mo_7_O_24_ and 20 μM FeEDTA; pH was adjusted to 4.8 with HCl. The total volume of the solution was kept constant by adding deionised water to compensate the water lost through plant transpiration, sampling and evaporation. The solutions were changed every 3 days and pH was adjusted daily.

After 21 days of treatment, 10 plants were harvested for analysis. Shoots were separated from roots, rinsed three times with cold water and blotted between two layers of filter-paper. Roots were dipped in a 0.01 M HCl cold solution to eliminate external Ni or Cd adsorbed at the root surface according to Aldrich et al. (2003). Roots were then rinsed three times with cold distilled water and blotted with filter-paper. The xylem sap collection was performed on 10 plants per treatment at the end of a 3 week period. The shoots were excised 2 cm above the root and the solution exuded from the cut surface, after discharging the first drop, was considered xylem sap. Samples were collected by means of trapping into a 1.5 mL plastic vial filled with a small piece of cotton for 2 h after cutting. After determination of exuded volumes, the xylem sap samples were stored at -20^∘^C until analysis.

### Sample Preparation

Roots and shoots were frozen in liquid nitrogen and then freeze-dried. Samples were ground with a mortar and pestle to a fine powder and 50 mg samples were sequentially extracted with 4 mL HCL 0.1 N the mixture was centrifuged for 15 min at 15,000 *g.* The separated supernatant was further ultracentrifuged for 45 min at 15,000 *g* and the new supernatant was filtered throughout a 0.22 μm Millipore filter. The xylem saps also were filtered with sterile filters MILEX-GV of 0.22 μm (Millipore).

### Instrumentation and Analytical Procedures

Organic acid samples were analyzed using a Varian HPLC system with a tertiary gradient pump, a Gemini-NX reverse-phase HPLC column (100 × 4.5 mm; Phenomenex, Torrance, CA, USA), a variable wavelength UV/VIS detector and an autosampler equipped with a refrigerated sample compartment (Varian Canada Inc, Mississauga, ON, Canada). Samples were filtered across a Nalgene nylon membrane filter (0.45-μm diameter) supplied by Nalge Company (Rochester, NY, USA). The injected sample volume was 20 μL in the case of shoot and root extracts and 10 μL in the case of xylem sap. The organic acids were eluted with 0.008 N H_2_SO_4_ /H_2_O at 1.0 ml min^-1^ flow under isocratic conditions and monitored at 210 nm for malic, citric, and fumaric acids, and at 245 nm for ascorbic acid. The acid compounds were identified based on retention time and UV spectra relative to standards. A multilevel calibration method with daily prepared standard solutions was used for quantitative determination of the acids. Each sample was analyzed in triplicate.

Ten μL of the collected xylem saps were diluted in 5 mL of 0.1 N HNO_3_ before Cd and Ni (II) concentrations were determined by inductively coupled plasma-mass spectrometry by a Varian 820 ICP-MS. For all the measures by ICP-MS, an aliquot of 2 mg L^-1^ of an internal standard solution (^45^Sc, ^89^Y, ^159^Tb) was added both to samples and calibration curve to give a final concentration of 20 mg L^-1^. The instrument was tuned daily with a multi-element tuning solution for optimized signal-to-noise ratio.

### Statistical Analysis

ANOVA with orthogonal contrasts and mean comparison procedures were used to detect differences between treatments. Mean separation procedures were conducted using the multiple range tests with Fisher’s least significant difference (LSD; *P* < 0.05).

## Results

### Effects Metal Treatment on Plant Growth and Development

**FIGURE 1:**
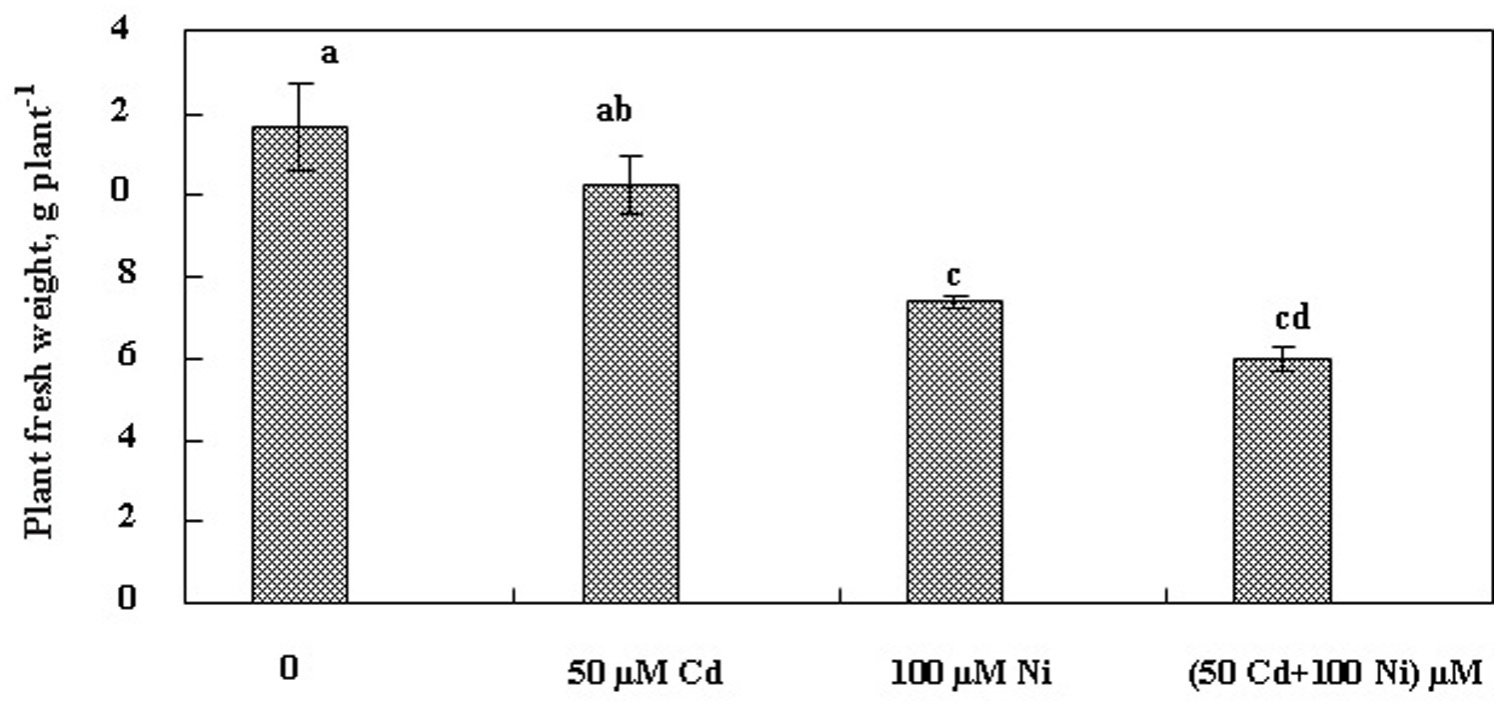
**Changes in fresh weights (FW; g plant^-1^ ) of *Sesuvium portulacastrum* subjected during 3 weeks to various treatments [(50 μM Cd, 100 μM Ni) and the combination of (50 μM Cd + 100 μM Ni )].** Means of eight replicates. Bars marked with same letter are not significantly different at *p* = 0.05.

The effect of Cd and Ni applied separately or together on *S. portulacastrum* development was evaluated based on fresh biomass production after 21 days of treatment. Our results (**Figure 1**) indicated that 50 μM Cd had no significant impact on the biomass production in this halophyte species. Hence under this treatment, the reduction of biomass production did not exceed 25% as compared to control plants. In contrast, Ni alone as well as the combination of both metals (Cd + Ni) significantly reduced plant growth. This biomass reduction reached 35 and 49% as compared to control respectively under Ni and Cd + Ni treatments.

### Metals Concentrations in Root, Shoot, and Xylem Sap

**FIGURE 2:**
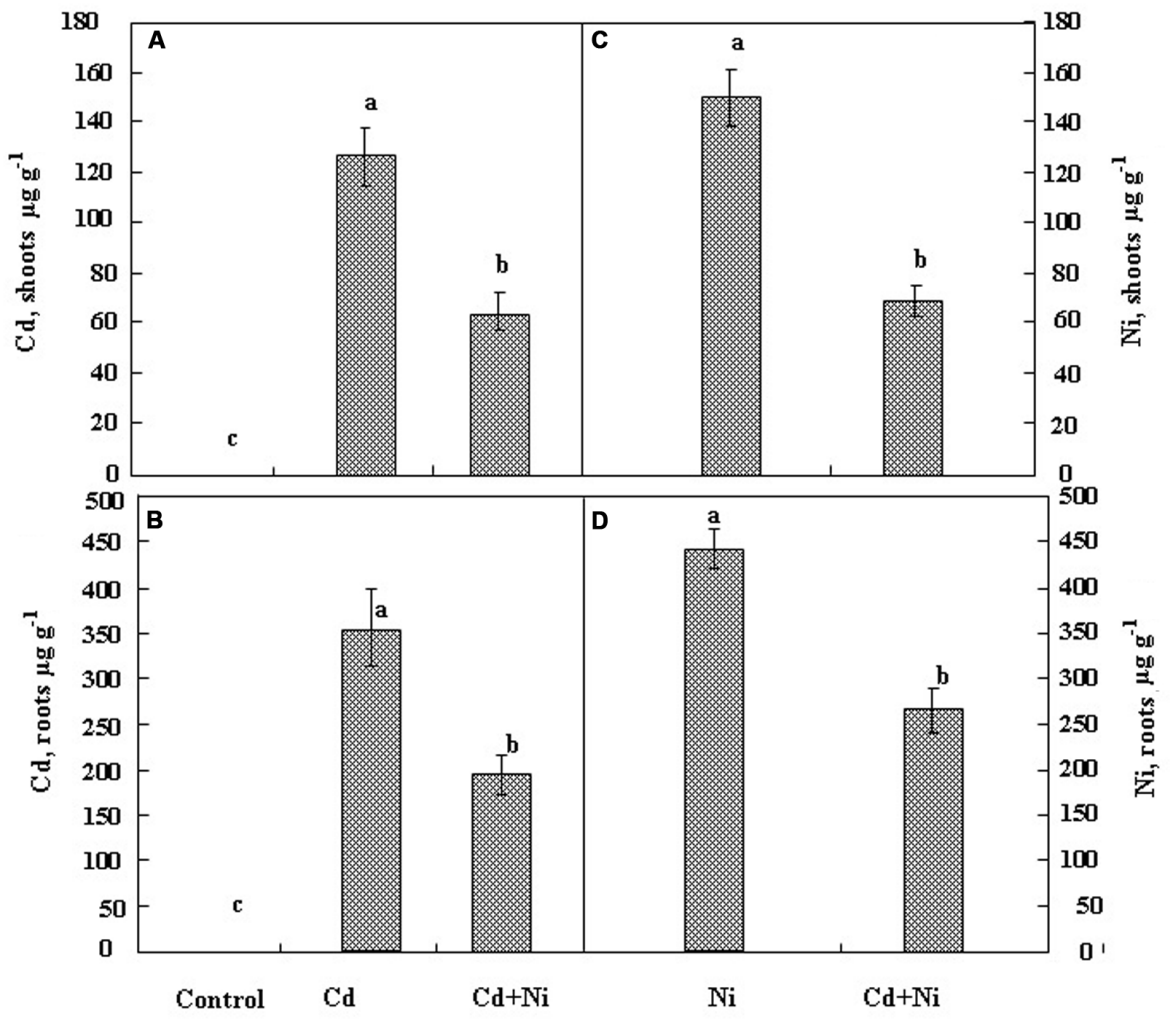
**Variation in Cd concentrations in shoots **(A)** and roots **(B)** and Ni concentrations in shoots **(C)** and roots **(D)** in *S. portulacastrum* cultivated during 21 days under different treatments: control without metal; 50 μM Cd; 100 μM Ni and the combination of (50 μM Cd + 100 μM Ni).** Means of eight replicates. Bars marked with same letter are not significantly different at *p* = 0.05.

The variation of Cd and Ni concentrations in the shoots and the roots of *S. portulacastrum* cultivated during 21 days in the presence of 50 μM Cd and 100 μM Ni applied separately or together are given in **Figure 2**.

Results showed that both metals accumulated to a higher extent in the roots than in the shoots. It is noteworthy that the combination of Cd and Ni in the nutrient solution significantly reduced the accumulation of Cd and Ni inside the root and the shoot tissues. Hence metal (Cd or Ni) concentrations in tissues under combined treatments represented only 50% of those measured when the metal (Cd or Ni) were added separately to the nutrient solution. Such a behavior is in the favor of putative competition between Cd^2+^ and Ni^2+^ for the absorption process at the root cell plasma membrane.

**FIGURE 3:**
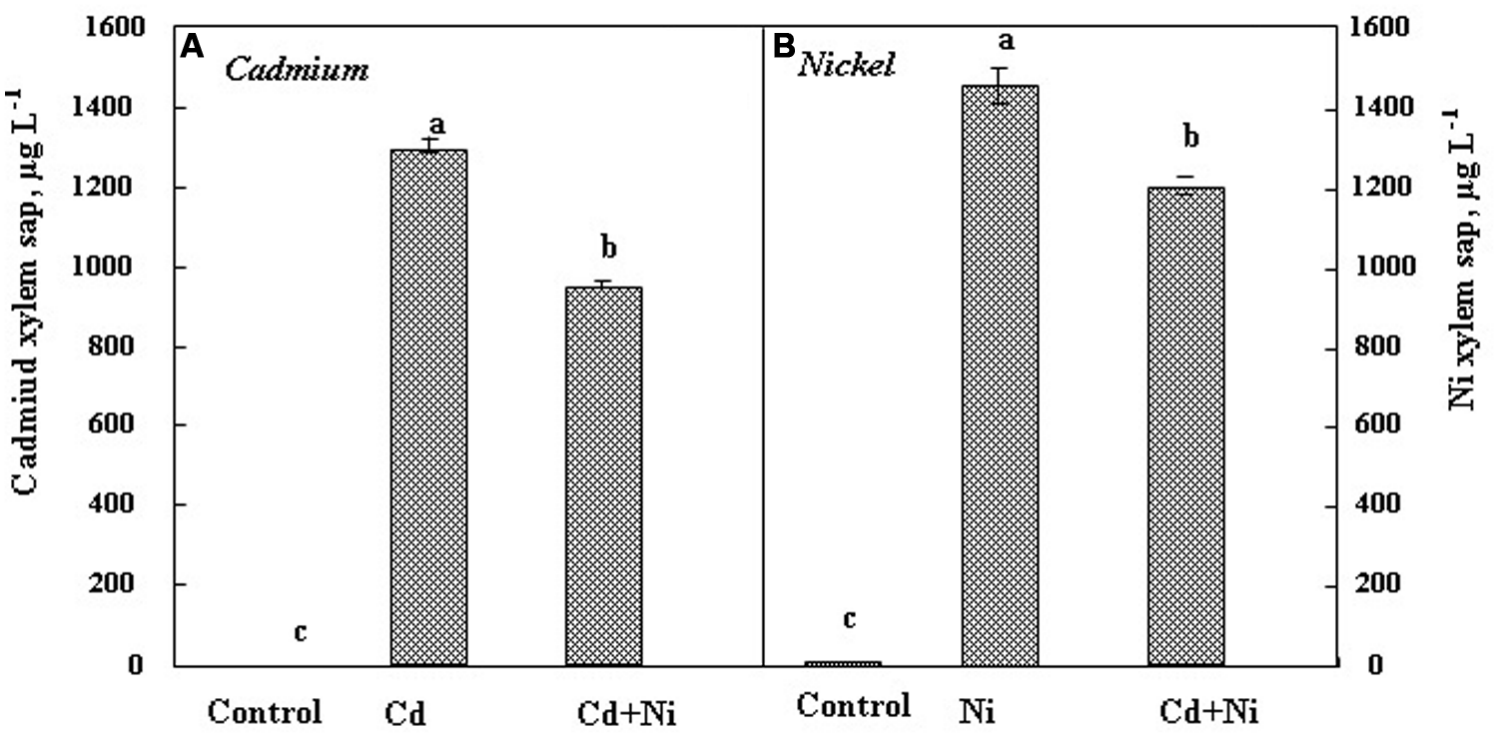
**Cadmium **(A)** and Nickel **(B)** concentrations in xylem saps of *S. portulacastrum* subjected during 21 days to different treatments.** Means of eight replicates. Bars marked with same letter are not significantly different at *p* = 0.05.

In the xylem sap (**Figure 3**), Cd and Ni were more concentrated than in the shoot both in individual and combined treatments. Nevertheless, as reported for leaves and roots, the simultaneous presence of Cd and Ni reduced the metal concentration in the ascendant sap. Nickel was more concentrated in the xylem sap as compared to Cd. The Cd and Ni concentrations in xylem sap of *S. portulacastrum* exposed to 50 μM Cd and 100 μM Ni were 1300 and 1450 μg L^-1^, respectively. These results confirm the high potential of this halophyte not only to absorb, but also to translocate several metals from roots toward the shoots.

### Modification of Organic Acid Concentrations in Tissues Under Different Treatments

**FIGURE 4:**
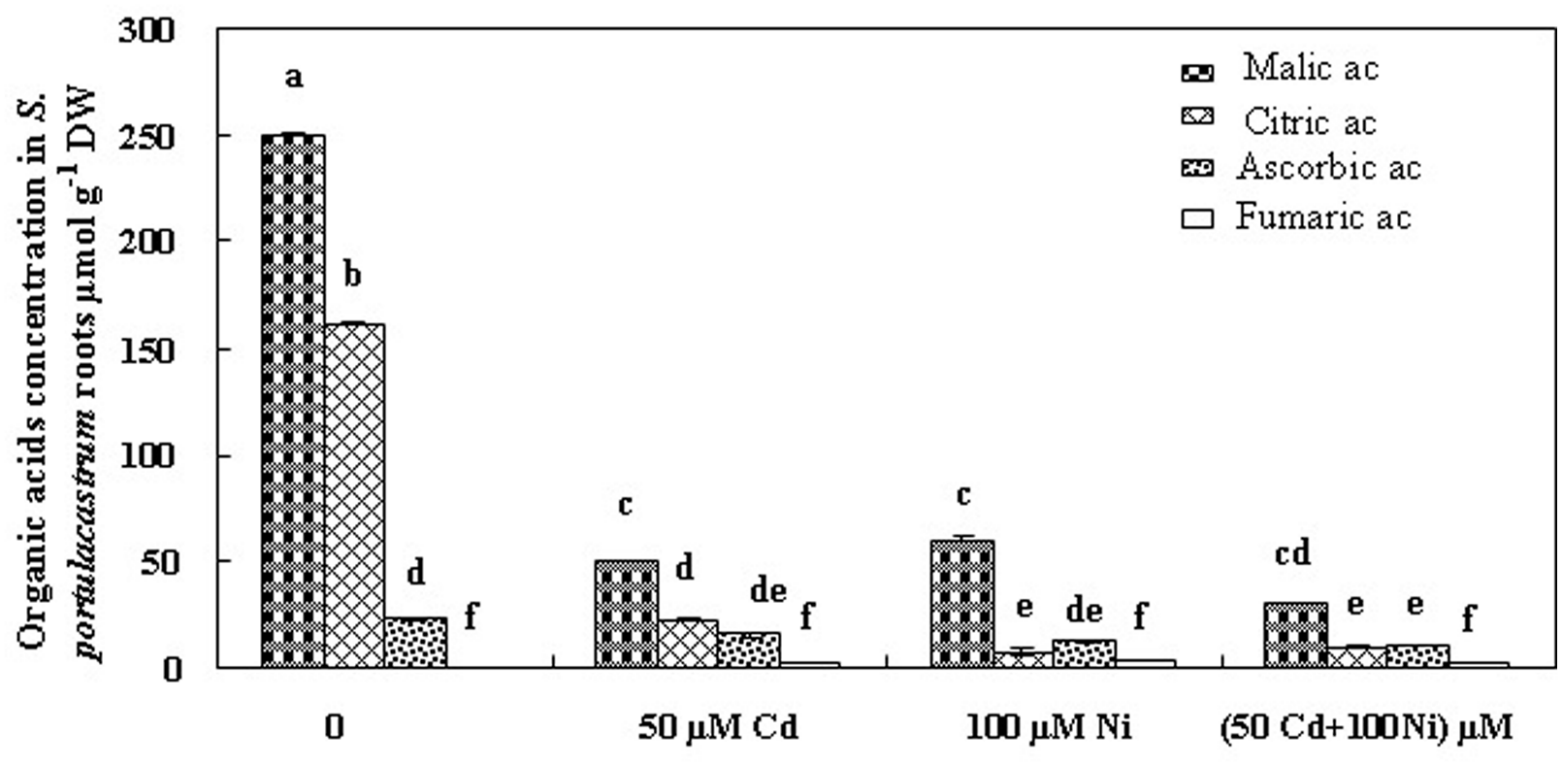
**Concentrations of organic acids in roots of *S. portulacastrum* subjected during 21 days to different treatments.** Means of eight replicates. Bars marked with same letter are not significantly different at *p* = 0.05.

**FIGURE 5:**
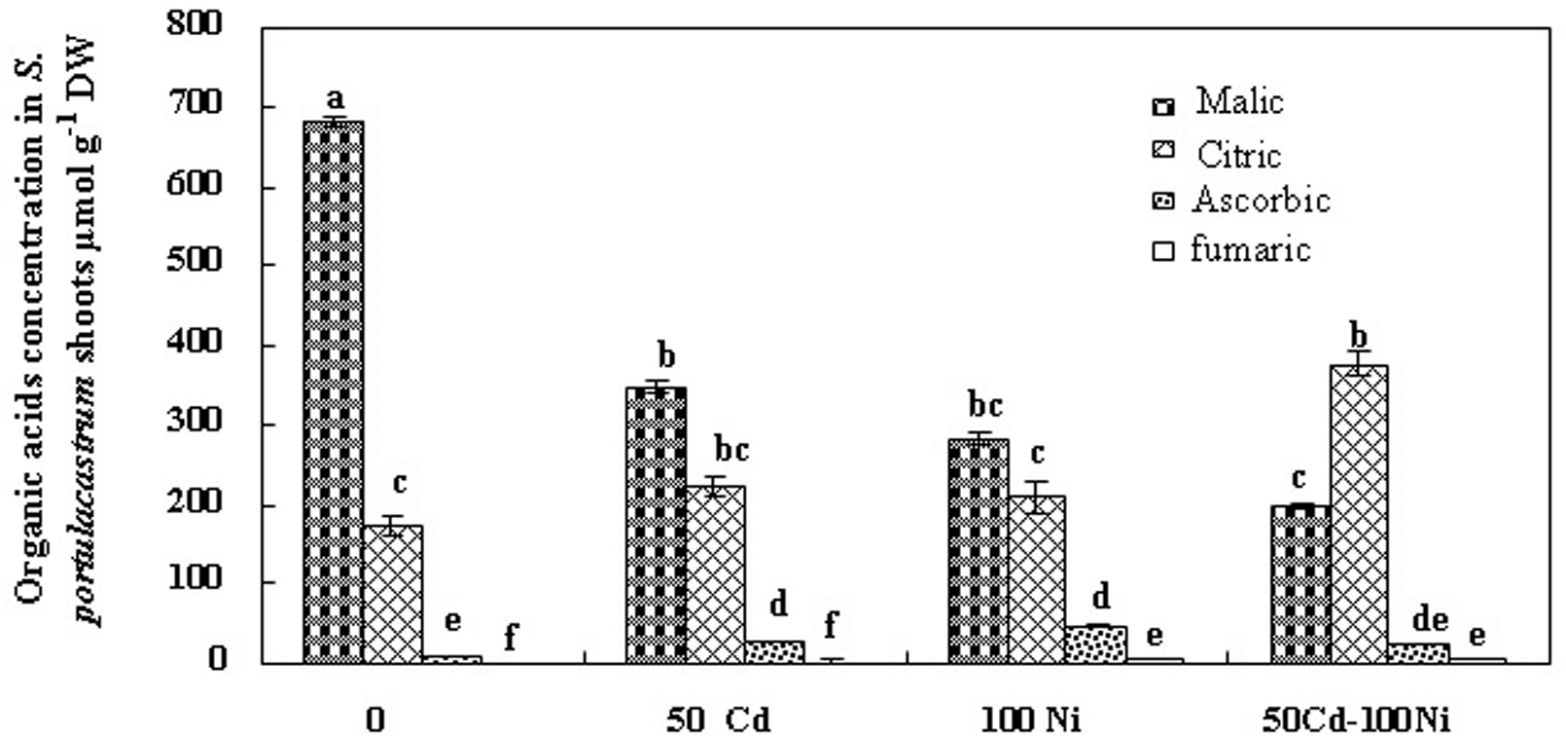
**Concentrations of organic acids in shoots of *S. portulacastrum* grown in control and subjected during 21 days at different metal treatments.** Means of eight replicates. Bars marked with same letter are not significantly different at *p* = 0.05.

In order to evaluate the possible implication of organic acids in Cd and Ni, translocation and/or chelation and tolerance in *S. portulacastrum*, we estimated the concentrations of these compounds in tissues of plant cultivated under different treatments (**Figures 4 and 5**). Fumarate, ascorbate, citrate, and malate were the major organic acids detected. Our analysis demonstrated that malic and citric acids were the most abundant organic acids in the roots and the shoots of this halophyte under different Cd and Ni combination (**Figures 4 and 5**). Hence due to the low modification in the fumaric and ascorbic acids contents in tissues of plants exposed to metal stress as compared to controls, we focused hereafter on the variation of malic and citric acids concentrations.

The addition of 50 μM Cd and 100 μM Ni alone or in combination reduced the malic acid concentration in the roots (**Figure 4**) and in the shoots (**Figure 5**) of *S. portulacastrum*. This reduction was of the same amplitude for Cd and Ni when applied separately but was accentuated in response to a combined application. These data suggest that heavy metals inhibited the biosynthesis of malic acid**.**

Citric acid is the first metabolite synthesized by the Krebs cycle. We demonstrated that citric acid concentration was reduced in the roots of plants subjected to Cd, Ni, and Cd + Ni (**Figure 4**). In contrast, the presence of both metals together or separately in the culture medium induced a significant increase of citrate concentrations in the shoot (**Figure 5**). This effect was more obvious under combined Cd + Ni treatment since the relative increase compared to control plants was 55, 93 and 258% under Cd, Ni, and Cd + Ni treatments, respectively.

**FIGURE 6:**
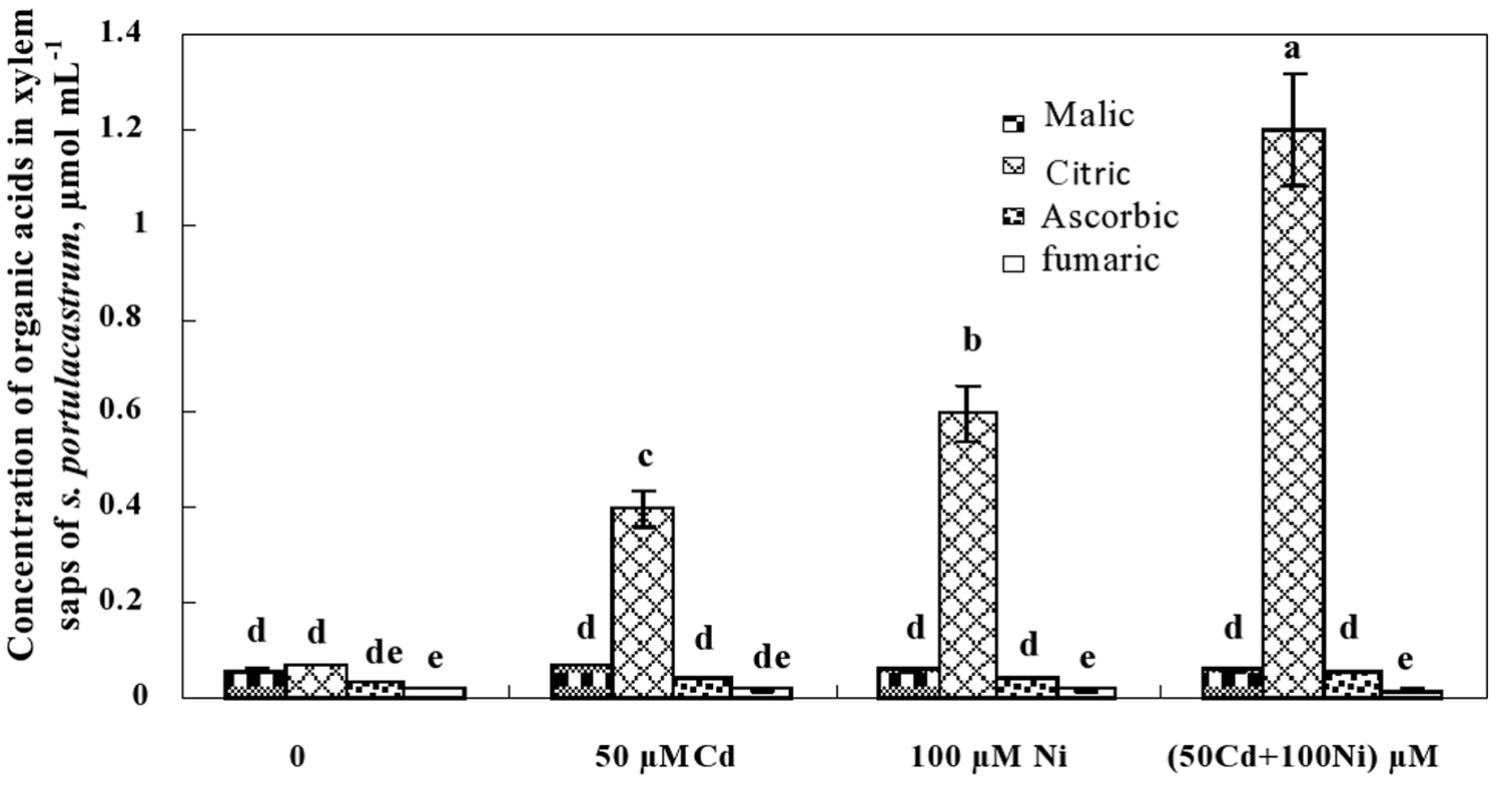
**Concentrations of organic acids in xylem saps of *S. portulacastrum* grown during 21 days on control and metal enriched solutions.** Means of eight replicates. Bars marked with same letter are not significantly different at *p* = 0.05.

This preliminary data showing the decrease of citrate concentration in the roots and its increase in the shoots suggests the possible implication of this carboxylic acid in the translocation of metal ions (Cd^2+^ and Ni^2+^) from the roots to the shoots through the xylem vessels. The determination of organic acid concentrations in the xylem sap (**Figure 6**) showed that malic, ascorbic, and fumaric acids were detectable in the xylem sap of control and metal-treated *S. portulacastrum* plants. However, the reduced and unchanged concentration of malic, ascorbic, and fumaric acids in the xylem sap under control and metal treatments (**Figure 6**) suggest that these compounds are not involved in long distance transport of Cd and Ni in this species. In contrast, citric acid concentration drastically increased in the xylem sap of Cd and Ni-treated plants (**Figure 6**). The citric acid concentration in the xylem was metal dose-dependent and increased with increasing total metal concentration in the medium as follow: 50 μM Cd < 100 μM Ni < 50 μM Cd + 100 μM Ni.

## Discussion

Several recent works demonstrated that halophyte species are more adapted to cope with abiotic stress including heavy metals than salt sensitive ones (Lefèvre et al., 2010; Zaier et al., 2010; Amari et al., 2014; Taamalli et al., 2014). Hence it has been demonstrated that *S. portulacastrum*, for example, is able to accumulate Cd and Pb in the shoots without significant growth reduction (Ghnaya et al., 2005; Zaier et al., 2010). These authors and others (Amari et al., 2014; Taamalli et al., 2014) demonstrated that halophyte plants may tolerate heavy metals more efficiently than conventional glycophyte accumulator species such as *Brassica juncea*. This capacity may be controlled by several mechanisms of metal detoxification as the overproduction of phytochelatins (Zaier et al., 2010) and chelation to organic acids (Ghnaya et al., 2013). However, for plant responses to the combination of metals, only little data concerning halophyte species are available.

The tolerance to heavy metals in plants could be controlled by two essential strategies: exclusion and accumulation. The exclusion one signifies that a plant avoids or restricts the absorption of metals while accumulation is directly related to the ability of the plant to sequester metals inside the tissues. In our study, we showed that *S. portulacastrum* is able to accumulate Cd and Ni which confirm the previous results published by Ghnaya et al. (2005, 2007) and Zaier et al. (2010, 2014) demonstrating that this species adopts the second strategy. However, we also showed that, the coexistence of both Cd and Ni in the medium reduced the concentration of Cd^2+^ and Ni^2+^ in the tissues of this halophyte suggesting a competitive interaction between both elements for the absorption through the root cell membranes. The competition between bivalent metal-cations for absorption at the cell membrane level was previously suggested (Ghnaya et al., 2007; Assuncão et al., 2008; Zaier et al., 2014) and was attributed to the low specificity of metal transporters and the chemical similarities between cations (Zhao et al., 2002; Caille et al., 2005).

Previous studies reported that *S. portulacastrum* is able to accumulate Cd, Pb, and As at levels to characteristic of accumulator plant species (Ghnaya et al., 2005, 2013; Zaier et al., 2010; Lokhande et al., 2011; Wali et al., 2014). Nevertheless, this study is the first one, to the best of our knowledge, to show that this halophyte is also able to accumulate 150 μg Ni g^-1^ DW in the shoots, strengthening the hypothesis that metal tolerance mechanisms selected by this species are not specific to one single element and may thus be used for phytoremedation of polymetallic contaminated soils.

With respect to metal translocation, many studies suggested that in the xylem vessel heavy metals are transported in complexed forms with different ligands (Rascio and Navari-Izzo, 2011). Organic acids have been described as potential chelators to facilitate Ni and Cd transport in some plant species (Sarret et al., 2002; Sun et al., 2006). However, the implication of organic acids in metal tolerance and cell accumulation is still under discussion. In fact, the global mechanisms of metal hyperaccumulation and detoxification in plants have not been fully elucidated yet and it is widely accepted that they rely on a multitude of interacting properties in plant. Several data suggest that chelation with specific organic acids constitutes an important procedure to efficiently transport and avoid the toxicity of free reactive metal ions in plants (Wei et al., 2007, 2009). For example, the Cd-hyperaccumulator *T. caerulescens* (syn. *Noccaea caerulescens*) synthesizes more organic acids when subjected to Cd^2+^ in order to reduce the reactivity of free Cd ions with proteins (Salt et al., 1999; Pence et al., 2000; Callahan et al., 2006).

The evaluation of the Pb translocation and accumulation in *S. portulacastrum* and the possible implication of organic acids in these processes were studied by Ghnaya et al. (2013). These authors demonstrated that this halophyte accumulated 1470 μg PbL^-1^ in its xylem sap when cultivated in the presence of 200 μM PbNO_3_*.* The Pb translocation in this species is facilitated by their chelation to malic and citric acids (Ghnaya et al., 2013).

However, our study showed that Cd and Ni reduced the biosynthesis of malate (**Figures 4 and 5**) when applied together or separately in this species. It is possible that fumarase activity responsible for conversion of fumarate to malate was inhibited. However, if this explanation is valid, then fumarate concentration should increase in tissues, which is not the case in this work. The second explanation, which is more logical and convincing, postulates that due to the excessive need in citric acid to chelate and transport Cd and Ni, disruption of the Krebs cycle leading to a deficit in malic acid may occur. Finally malate may also be excreted as exudates in the external medium (Mucha et al., 2010; Ghnaya et al., 2013).

On the other hand, the decrease of citrate concentration in the roots and its increase in the shoots suggests the possible implication of this carboxylic acid in the translocation of metal ions (Cd^2+^ and Ni^2+^) from the roots to the shoots through the xylem vessels. In the same context, the build-up in shoot citrate concentrations under different heavy metal exposure was observed in many plant species (Irtelli and Navari-Izzo, 2006; Sun et al., 2006; Ghnaya et al., 2013) and could be related to their tolerance and shoot accumulation traits (Krämer et al., 2000; Sun et al., 2006; Ghnaya et al., 2013). Also, we suggest that citric acid could be highly implicated in the Cd and Ni translocation from roots to the shoots in this species. We demonstrated here that the xylem sap of plants exposed to toxic metal was more concentrated in citrate than in control plants. The citrate concentration was also positively correlated with the xylem sap Cd + Ni concentration. Hence, the high potential of Cd and Ni translocation and shoot accumulation exhibited by *S. portulacastrum* could be related and governed by the higher citrate levels present in leaf cells as previously shown in studies on Pb translocation and accumulation (Zaier et al., 2010; Ghnaya et al., 2013).

## Conclusion

Taken together the results obtained in this work indicated that 50 μM Cd does not induce significant change in the growth of the halophyte *S. portulacastrum* which should be related to the tolerance of this species against some heavy metals. However, 100 μM Ni and the combination of both 50 μM Cd and 100 μM Ni significantly reduced plant growth. Our data suggest possible competition between Cd and Ni for root absorption. Among organic acids, ascorbic and fumaric acids showed the lowest concentrations in the xylem sap and remained unchanged under Cd and Ni application, while malic and citric acids showed significant modification in response to Cd and Ni. Malic acid concentration was reduced in roots and shoots of plants exposed to toxic metals but remained unchanged in the xylem sap. The citric acid concentration was reduced in roots of plants treated with Cd and Ni, while an opposite behavior was observed in the shoots and xylem sap. The positive correlation between Cd^2+^ + Ni^2+^ and citric acid xylem-sap concentrations strongly suggests the implication of citric acid in metal translocation.

In addition, the enhancement of this acid concentration in shoots is in favor of its possible implication in metal chelation and sequestration in these organs. The main results related to the organic acids concentration under metal stress indicate that citric acid could be directly involved in Cd and Ni translocation and accumulation in the shoots of this halophyte.

## Conflict of Interest Statement

The authors declare that the research was conducted in the absence of any commercial or financial relationships that could be construed as a potential conflict of interest.
